# Addressing Mental Health Priorities in Mexican Youth: Developing a Primary Health Care and Psychiatry Model (MAP-PSI for its acronym in Spanish)

**DOI:** 10.1192/j.eurpsy.2025.686

**Published:** 2025-08-26

**Authors:** L. Diaz-Castro, N. Bautista-Aguilar

**Affiliations:** 1 National Institute of Psychiatry Ramon de la Fuente, Mexico City, Mexico

## Abstract

**Introduction:**

Depressive disorders among young people are a significant public health concern, particularly in underserved areas where access to mental health services is limited. The MAP-PSI model was developed to address this gap through a comprehensive approach that integrates promotion, prevention, and remote care strategies. Implemented in Ciudad Fernández, San Luis Potosí, Mexico, the model is designed to be adaptable and scalable, allowing it to meet the varying needs of different communities and leverage telemedicine and digital tools to extend mental health care.

**Objectives:**

The MAP-PSI model aims to improve mental health outcomes for young people by providing an adaptable framework for addressing depressive disorders. Its goals are to enhance accessibility to mental health services in underserved areas, utilize bilingual and culturally sensitive materials, and integrate actions across various community settings. The model seeks to empower healthcare providers and decision-makers to implement effective, sustainable interventions that can be adapted for other health issues and locations.

**Methods:**

The MAP-PSI model is implemented in four phases:Exploration-Planning: This phase involves community engagement, conducting needs assessments, and training local staff to ensure that the intervention is tailored to the specific needs of the community. Initial and Total Implementation: Active monitoring and real-time adjustments are made to the model to ensure its effectiveness and responsiveness to emerging needs. Sustainability: Focuses on maintaining and expanding the model’s reach through continuous improvement processes, ensuring long-term impact and adaptation.

**Results:**

Key components of the model include psychoeducation, psychological support, telepsychiatry, and community-based promotion and prevention efforts. The use of bilingual and culturally sensitive materials is emphasized to cater to diverse populations, including indigenous groups. Integration across primary health care centers, schools, and community settings enhances the support for mental health and prevention of depressive disorders.

**Conclusions:**

The MAP-PSI model demonstrates promising social returns on investment by effectively addressing depressive disorders through a scalable and adaptable framework. Its integration of telemedicine and digital tools enhances accessibility to mental health care in underserved areas, while its culturally sensitive approach ensures relevance across diverse populations. The model’s phased implementation framework supports both immediate effectiveness and long-term sustainability. The MAP-PSI model offers a valuable guide for health care providers and decision-makers, with the potential for adaptation and expansion to other health issues and regions, thereby contributing to improved mental health outcomes and sustainable intervention practices.

**Disclosure of Interest:**

None Declared

